# Analytical wave families and stability dynamics in a modified complex Ginzburg–Landau model via the modified extended direct algebraic method

**DOI:** 10.1038/s41598-026-37824-0

**Published:** 2026-02-21

**Authors:** Adel E. Rateb, Hamdy M. Ahmed, Adel Darwish, Medhat Ammar, Wafaa B. Rabie

**Affiliations:** 1 Department of Mathematics, Faculty of Science, Helwan University, Cairo, Egypt; 2 Department of Computer Science, Higher Institute for Computers and Information Technology, El Shorouk Academy, Cairo, Egypt; 3 Department of Physics and Engineering Mathematics, Higher Institute of Engineering, El Shorouk Academy, Cairo, Egypt; 4 Department of Mathematics, Faculty of Science, Luxor University, Taiba, Luxor, Egypt

**Keywords:** Nonlinear dynamics, Exact solutions, Solitons, Stability analysis, Wave propagation, Mathematics and computing, Physics

## Abstract

This study investigates the Modified Complex Ginzburg–Landau Equation, a fundamental nonlinear partial differential equation that plays a central role in modeling complex wave dynamics, pattern formation, and dissipative phenomena in systems such as nonlinear optics, Bose–Einstein condensates, superfluids, and plasmas. Despite its importance, obtaining exact analytical solutions and understanding their stability properties remain challenging problems with significant theoretical and practical implications. To address this challenge, the Modified Extended Direct Algebraic Method is employed to construct exact analytical solutions in a systematic and efficient manner. By transforming the governing nonlinear equation into an algebraically solvable system, a broad and unified family of exact solutions is derived. These solutions include bright and dark solitons, singular solutions, periodic and singular periodic waves, as well as solutions expressed in exponential, Weierstrass elliptic, and Jacobi elliptic function forms. In addition, a comprehensive stability analysis is carried out to examine the response of these wave structures to small perturbations and to assess their long-term dynamical behavior. The physical characteristics and dynamical features of the obtained solutions are illustrated through detailed two-dimensional and three-dimensional graphical representations for selected parameter values. The results demonstrate the effectiveness of the Modified Extended Direct Algebraic Method in analyzing complex nonlinear models and provide deeper insight into wave propagation and stability mechanisms in dissipative systems governed by the Modified Complex Ginzburg–Landau Equation.

## Introduction

Nonlinear partial differential equations (NLPDEs) play a pivotal role in modeling complex physical phenomena characterized by nonlinear interactions, wave propagation, and pattern formation. Their significance stems from their ability to mathematically describe intricate dynamical behaviors across diverse scientific domains, including optics, fluid dynamics, plasma physics, and quantum mechanics^1–5^. Among the most remarkable solutions to NLPDEs are solitons–stable, localized waves that maintain their shape and velocity over long distances. These waves have revolutionized modern science, particularly in optical fiber communications, where they enable high-speed data transmission with minimal distortion^6–11^. Beyond telecommunications, solitons are instrumental in oceanography (modeling tsunamis and rogue waves), astrophysics (describing magnetospheric and solar wind dynamics), and plasma physics, where they help predict space weather phenomena^12–14^. Extensive recent studies have further explored soliton dynamics in various fractional and conformable models^15–17^. A cornerstone in soliton theory is the nonlinear Schrödinger equation (NLSE), which governs wave dynamics in dispersive and nonlinear media. Extensive research has been devoted to solving the NLSE using advanced analytical and numerical techniques. For instance, Wazwaz and Mehanna^18^ derived bright and dark solitons for the (3+1)-dimensional NLSE, while Rabie et al.^19^ investigated higher-order dispersive effects in NLSE with cubic-quintic nonlinearity. Kudryashov^20^ and Rezazadeh et al.^21^ explored resonant and non-autonomous NLSE variants, respectively, uncovering novel soliton structures. Akinyemi et al.^22^ and Rabie et al.^23^ further extended these studies to perturbed and highly dispersive NLSE systems, demonstrating the equation’s versatility. Other breakthroughs include coupled NLSE systems^24–26^, magneto-optic waveguides^27^, and (3+1)-dimensional chiral NLSE models^28^, each revealing new facets of nonlinear wave behavior. Additionally, Bilal et al.^29^ recently explored the dynamical analyses and dispersive soliton solutions to nonlinear models in stratified fluids, further extending the understanding of wave structures in complex media. Recent advancements also include studies on fractional and conformable models, such as the fractional Landau-Ginzburg-Higgs equation^30^ and space-time fractional modified Benjamin-Bona-Mahony equation^17,31,32^. The quest for novel solutions has also been driven by innovative methodologies. For instance, Muhammad et al.^33,34^ applied neural network-based symbolic methods to analyze nonlinear dynamical equations, while Hussain et al.^35,36^ explored optical multi-peakon dynamics using fractional models and novel integral approaches. Furthermore, the application of advanced analytical techniques, such as the modified extended direct algebraic method (mEDAM)^30,32,37^, the Sardar-subequation method^26^, and the extended direct algebraic method^17,38^, has yielded a rich variety of soliton solutions for complex fractional and integer-order systems. Studies on the dynamics of kink solitons in fractional Kolmogorov–Petrovskii–Piskunov equations^39,40^ and the exploration of soliton structures in fractional Heisenberg ferromagnetic spin chains^31^ further underscore the depth of current research. The analysis of thermophoretic motion through graphene sheets^29,41^ and the investigation of wave dynamics in higher-dimensional nonlinear evolution equations^42^ highlight the interdisciplinary applications of these methods. Ginzburg and Landau introduced a paradigm-shifting model to describe superconductivity and superfluidity–the complex Ginzburg-Landau equation (CGL). This equation balances nonlinear self-interaction and linear dispersion, enabling the study of solitons, turbulence, and pattern formation in dissipative systems^43–45^. A generalized variant, the modified complex Ginzburg-Landau equation (MCGL), incorporates additional nonlinear and dispersive terms, making it indispensable for modeling plasma turbulence, dissipative solitons in lasers, and pattern formation in fluids^46–48^. Unlike conservative systems, the MCGL equation accounts for energy dissipation and gain, offering a more realistic framework for physical systems with inherent losses. Furthermore, recent study by Ismael et al.^35^ demonstrated soliton-like and hybrid thermophoresis behavior in optical contexts, emphasizing the role of such nonlinear models in characterizing motion through graphene sheets. Additionally, several studies have investigated optical solitons and wave phenomena in various Ginzburg-Landau models^42,49–51^. Significant contributions have been made in obtaining analytical solutions for the CGL and MCGL equations. For example, Zafar et al.^49,50^ employed modified expansion schemes to extract optical solitons for the nonlinear CGL equation. Yomba and Kofané^46^ and Hong^48^ derived exact and stable stationary soliton solutions for the one-dimensional MCGL equation, respectively. More recently, Raza et al.^47^ performed a dynamic analysis and derived new optical soliton solutions for the MCGL model in communication systems, while Zhu et al.^45^ investigated bifurcations, chaotic behavior, and optical solutions for the CGL equation. These efforts highlight the ongoing relevance and challenge of solving this class of equations. The modified complex Ginzburg-Landau equation (MCGL) stands as one of the most significant models in nonlinear science, bridging the gap between theoretical physics and real-world wave dynamics. Its mathematical framework is crucial for modelling a vast array of physical phenomena, particularly in systems far from equilibrium. Recent advancements in mathematical modelling of nonlinear systems underscore a paradigm shift towards analyzing higher-dimensional and more complex structures to capture realistic multi-scale interactions. For instance, symmetry analyses and comprehensive dynamical studies on various (3+1)-dimensional nonlinear models^52–56^ have not only provided powerful analytical and numerical methodologies but have also fundamentally enhanced our understanding of wave propagation, coherent structure formation, and energy localization in complex media. These studies exemplify how sophisticated mathematical models serve as indispensable tools for translating intricate physical laws into a quantifiable and predictive formalism. Despite its profound importance in describing dissipative solitons, optical turbulence, and pattern formation, obtaining exact analytical solutions for the MCGL equation remains a formidable challenge due to its intricate balance of nonlinearity, dispersion, and dissipation. The methodologies from these studies can be adapted to tackle such challenging equations. Furthermore, studies on bifurcation analysis, chaos, and sensitivity in nonlinear systems^15,57,58^ offer insightful frameworks for examining the complex behavior inherent in the MCGL equation. Highlighting the critical role of mathematical analysis in unraveling the transition between order and disorder, and predicting system behavior under parametric perturbations–a core aspect of nonlinear physical modelling. The governing model for this research is the modified complex Ginzburg-Landau equation (MCGL), expressed as^59,60^:

1 $$\begin{aligned} i \ V_{t} + \alpha \ V_{xx} + \beta \ V \ | V| ^2 -i \ \rho \ V - r \ \frac{V_{x} V_{x}^* }{V^*}- \gamma \ \left[ \frac{1}{2} V V^* \frac{\partial ^2\left( V V^*\right) }{\partial x^2}-\frac{1}{4} \left( \frac{\partial \left( V V^*\right) }{\partial x}\right) ^2\right] \frac{1}{V \left( V^*\right) ^2}=0, \end{aligned}$$

where the complex-valued function *V*(*x*, *t*) describes the wave envelope’s amplitude and phase evolution across spatial dimension *x* and temporal dimension *t*. The model incorporates several physically significant parameters: $\alpha$ (group velocity dispersion), $\beta$ (Kerr nonlinearity coefficient), $\rho$ (gain/damping parameter), *r* (complex mode coupling), and $\gamma$ (higher-order dispersion)—all taking real values.

Existing literature, notably in^60^, has explored the modified complex Ginzburg-Landau (MCGL) equation using an extended modified auxiliary equation mapping technique, deriving basic trigonometric, hyperbolic, and exponential soliton solutions. The present study significantly advances these analytical efforts by implementing the modified extended direct algebraic method (MEDAM). This methodological shift is not merely incremental; it is pivotal for several reasons. First, the MEDAM framework provides a more systematic and generalizable algebraic structure for handling the equation’s higher-order nonlinear and dispersive terms, effectively reducing them to an integrable form. This allows for the derivation of a *much broader and richer spectrum of exact solutions* than previously reported. Consequently, our work uncovers multiple novel solution classes for the MCGL equation that were unattainable with prior techniques. These include bright and dark solitons, singular solitons, periodic wave solutions, and more sophisticated waveforms expressed in terms of Jacobi and Weierstrass elliptic functions. *This constitutes the core novelty of our work:* the application of MEDAM to this specific, modified model yields a more comprehensive family of solutions, providing deeper insight into the nonlinear wave dynamics it governs, unlike the limited profiles obtained from simpler models or standard solution methods. Beyond analytical derivation, we augment our findings with comprehensive linear stability analysis and detailed 2D/3D graphical visualizations. These tools elucidate the physical characteristics, dynamic evolution, and stability regimes of the newly discovered solutions. The superior capability of MEDAM in managing complexity delivers accurate analytical descriptions, which in turn offer crucial insights into wave propagation stability and other nonlinear phenomena. Therefore, the *utility and originality* of this research are twofold: (1) It provides a substantial expansion of the known analytical solution space for the MCGL equation through a powerful and systematic method, and (2) it connects these theoretical advancements to practical relevance. The enriched set of solutions and their stability profiles significantly enhance the theoretical toolkit for modeling complex wave behavior in fields where the MCGL equation is paramount, such as nonlinear optics, condensed matter physics, and plasma science. The manuscript is structured to present these developments systematically: Section “Methodological foundation of the modified extended direct algebraic method” elucidates the theoretical framework of MEDAM; Section “Novel wave structures for the proposed equation” details the derivation of exact solutions; Section “Modulational instability analysis in the MCGLE” examines stability properties; Section “Result and discussion” provides graphical analysis of solution behaviors; and Section “Conclusion” summarizes principal findings and suggests future research directions.

## Methodological foundation of the modified extended direct algebraic method

The modified extended direct algebraic method (MEDAM) provides a systematic approach for obtaining exact solutions to nonlinear partial differential equations (NLPDEs). This powerful technique transforms complex nonlinear problems into solvable algebraic systems through an optimized solution ansatz^61,62^.

Let us consider a nonlinear partial differential equation (PDE) expressed as:

2 $$\begin{aligned} W (K,\ K_{t},\ K_{x_1}, \ K_{x_1 x_2},\ K_{tx_1},\ K_{t x_2} , ...)= 0, \end{aligned}$$

where *W* is a polynomial in *K* and its partial derivatives. The solution procedure follows these systematic steps:

1. Let
  3 $$\begin{aligned} K(t,\ x_1,\ x_2,\ x_3,..., \ x_n) = \mathbb {C}(\delta ) , \hspace{1cm} \delta = x - \vartheta \ t, \end{aligned}$$
  where $\vartheta \ne 0$ is a constant wave velocity. Applying this transformation to the original PDE (2), we obtain the equivalent ordinary differential equation (ODE) as follows.
  The traveling wave transformation reduces all independent variables into a single composite variable $\delta$. Consequently, partial derivatives transform into ordinary derivatives via the chain rule:
  $$ \frac{\partial K}{\partial t} = -\vartheta \, \mathbb {C}', \qquad \frac{\partial K}{\partial x} = \mathbb {C}', \qquad \frac{\partial ^2 K}{\partial x^2} = \mathbb {C}'', \quad \text {etc.} $$
  Substituting these expressions into Eq. (2) converts the PDE into an ODE where the only independent variable is $\delta$. This yields:
  4 $$\begin{aligned} \mathbb {N}[\mathbb {C},\ -\vartheta \ \mathbb {C}',\ \mathbb {C}'',...] = 0, \end{aligned}$$
  where $\mathbb {N}$ represents a nonlinear polynomial function in $\mathbb {C}$ and its successive derivatives with respect to $\delta$.
2. We assume the general form of the solution using the Modified Extended Direct Algebraic Method (mEDAM) can be expressed as a finite Laurent series:
  5 $$\begin{aligned} \mathbb {C}(\delta )=\sum _{n=-M}^M s_n \ W(\delta )^n, \end{aligned}$$
  where the function $W(\delta )$ satisfies the first-order auxiliary differential equation:
  6 $$\begin{aligned} W'(\delta ) = \sqrt{ m_0 + m_1 W(\delta )+ m_2 W(\delta )^2 + m_3 W(\delta )^3 + m_4 W(\delta )^4 + m_6 W(\delta )^6 }. \end{aligned}$$
  Here, $m_i$ (for $i = 0, 1, 2, 3, 4, 6$) are real constants that parameterize the auxiliary equation. Different sets of these constants generate distinct families of solutions for $W(\delta )$ (e.g., trigonometric, hyperbolic, elliptic, or rational functions). The appropriate set is not chosen arbitrarily; it is determined algorithmically by substituting the series (5) and the condition (6) into the ODE (4). This substitution yields a system of algebraic equations in the unknowns $s_n$, $m_i$, $\vartheta$, and other parameters. Solving this system simultaneously determines the specific values of $m_i$ that lead to consistent, non-trivial solutions for $\mathbb {C}(\delta )$, thereby selecting the physically and mathematically admissible solution families.
3. Using different possible values for $m_{0}, m_{1}, m_{2}, m_{3}, m_{4}, m_{6}$, yields various types of solutions as follow:
  **Set(1)**: $$m_{0}= m_{1}= m_{3}=m_{6}=0$$,
  $$\begin{aligned} W(\delta )=\sqrt{-\frac{m_{2}}{m_{4}}} {{\,\textrm{sech}\,}}[\delta \ \sqrt{m_{2}}],\hspace{0.3cm} m_{2}>0, m_{4}<0. \end{aligned}$$
  $$\begin{aligned} W(\delta )=\sqrt{-\frac{m_{2}}{m_{4}}} \sec [\delta \ \sqrt{-m_{2}}],\hspace{0.3cm} m_{2}<0, \ m_{4}>0. \end{aligned}$$
  $$\begin{aligned} W(\delta )=\sqrt{-\frac{m_{2}}{m_{4}}} \ \csc [\delta \ \sqrt{-m_{2}}],\hspace{0.3cm} m_{2}<0, \ m_{4}>0. \end{aligned}$$
  **Set(2)**: $$m_{3}= m_{4}= m_{6}=0$$,
  $$\begin{aligned} W(\delta )=\frac{m_{1}\sinh [2\ \delta \ \sqrt{m_{2}}]}{2\ m_{2}}-\frac{m_{1}}{2\ m_{2}},\hspace{0.3cm} m_{2}>0,\ m_{0}=0. \end{aligned}$$
  $$\begin{aligned} W(\delta )=\frac{m_{1}\sin [\delta \ \sqrt{-m_{2}}]}{2\ m_{2}}-\frac{m_{1}}{2\ m_{2}},\hspace{0.3cm} \ m_{2}<0,\ m_{0}=0. \end{aligned}$$
  $$\begin{aligned} W(\delta )=\sqrt{\frac{m_{0}}{m_{2}}}\ \sinh [\delta \ \sqrt{m_{2}}],\hspace{0.3cm} m_{0}>0, \ m_{2}>0,\ m_{1}=0. \end{aligned}$$
  $$\begin{aligned} W(\delta )=\sqrt{-\frac{m_{0}}{m_{2}}}\ \sin [\delta \ \sqrt{-m_{2}}] ,\hspace{0.3cm} \ m_{0}>0,\ m_{2}<0,\ m_{1}=0. \end{aligned}$$
  $$\begin{aligned} W(\delta )=exp[\delta \ \sqrt{m_{2}}]-\frac{m_{1}}{2\ m_{2}},\hspace{0.3cm} m_{2}>0,\ m_{0}=\frac{m_{1}^{2}}{4\ m_{2}}. \end{aligned}$$
  **Set(3)**: $$m_{0}= m_{1}= m_{2}= m_{6}=0$$,
  $$\begin{aligned} W(\delta )=\frac{4\ m_{3}}{m_{3}^{2}\ \delta ^{2}-4\ m_{4}}. \end{aligned}$$
  **Set(4)**: $$m_{0}= m_{1}=m_{6}=0$$,
  $$\begin{aligned} W(\delta )=-\frac{m_{2} \left[ \tanh \left( \frac{\delta \ \sqrt{m}_{2} }{2}\right) +1\right] }{m_{3}} , \hspace{0.2cm} m_{2}>0. \end{aligned}$$
  $$\begin{aligned} W(\delta )=-\frac{m_{2} \left[ \coth \left( \frac{\delta \ \sqrt{m}_{2}}{2}\right) +1\right] }{m_{3}} ,\hspace{0.2cm}\ m_{2}>0. \end{aligned}$$
  **Set(5)**: $$m_{0}= m_{1}= m_{6}=0$$,
  $$\begin{aligned} W(z)=\frac{m_2 \ {{\,\textrm{sech}\,}}^2\left[ \frac{\delta \ \sqrt{m_2} }{2}\right] }{2 \sqrt{m_2 \ m_4} \ \tanh \left[ \frac{\delta \ \sqrt{m_2} }{2}\right] - m_3}. \end{aligned}$$
  **Set(6)**: $$m_{1}= m_{3}= m_{6}=0$$,

**Table:** 

No	$m_{0}$	$m_{2}$	$m_{4}$	$W(\delta )$
1	1	$-(1+r^{2})$	$r^{2}$	$cd(\delta ,r)$ or $sn(\delta ,r)$
2	$r^{2}$	$-r^{2}+1$	1	$ns(\delta ,r)$ or $dc(\delta , r)$
3	$r^{2}-1$	$2-r^{2}$	$-1$	$dn(\delta ,r)$
4	$\frac{r^{2}-1}{4}$	$\frac{r^{2}+1}{2}$	$\frac{r^{2}-1}{4}$	$r\ sd(\delta ,r)+nd(\delta ,r)$
4. The positive integer M is determined by balancing the highest-order derivatives with nonlinear terms in (4).
5. Substituting (5)–(6) into (4) yields a polynomial in W whose coefficients, when set to zero, form a nonlinear system solved symbolically using Mathematica to obtain the unknown parameters.

## Novel wave structures for the proposed equation

The traveling wave solution of (1) is defined as follows:

7 $$\begin{aligned} V = G(\delta ) \ e^{i \left( a x-b t+c_0\right) }, \end{aligned}$$

and

8 $$\begin{aligned} \delta =x-\lambda \ t, \end{aligned}$$

where $G(\delta )$ represents the wave amplitude envelope (real-valued function), $\delta$ is the traveling wave coordinate, *a* is the wave number, *b* is the angular frequency , $c_0$ is the initial phase constant, and $\lambda$ is the wave propagation speed.

Substituting the traveling wave solution (7) into the MCGL Eq. (1) yields:

9 $$\begin{aligned} (b - r a^2 -\alpha a^2) G(\delta )+(\alpha -\gamma ) G''(\delta )- r \ \frac{ G'(\delta )^2}{G(\delta )}+\beta \ G(\delta )^3+i [(\lambda -2 a \alpha ) G'(\delta )+\rho \ G(\delta )] = 0 . \end{aligned}$$

Separating the real and imaginary components in Eq. (9) leads to:

Real Part:

10 $$\begin{aligned} (b - r a^2 -\alpha a^2) G(\delta )+(\alpha -\gamma ) G''(\delta )- r \ \frac{ G'(\delta )^2}{G(\delta )}+\beta \ G(\delta )^3 = 0 . \end{aligned}$$

Imaginary Part:

11 $$\begin{aligned} (\lambda -2 a \alpha ) G'(\delta )+\rho \ G(\delta ) = 0 . \end{aligned}$$

Substituting (11) into (10) produces the simplified nonlinear ODE:

12 $$\begin{aligned} G''(\delta ) +\left(b- r \ a^2 -\alpha \ a^2 - \frac{\rho ^2 \ r}{(2 a \ \alpha -\lambda )^2}\right) \frac{G(\delta )}{\alpha -\gamma } +\frac{\beta \ \ G(\delta )^3 }{\alpha -\gamma } = 0 , \end{aligned}$$

Applying the homogeneous balance principle between $G''(\delta )$ and $G(\delta )^3$:

$$\begin{aligned} M+2=3\ M \rightarrow M=1. \end{aligned}$$

Then the balanced solution takes the form:

13 $$\begin{aligned} G(\delta )= \frac{s_{-1}}{W(\delta )}+s_0 +s_1 \ W(\delta ) , \end{aligned}$$

The values of the constants $s_{-1},\ s_0$, and $s_1$ are subsequently determined using Mathematica for solving the nonlinear system of equations, with the condition that either $s_{-1} \ne 0 \ or \ s_1 \ne 0$.

Subsequently, by substituting the expressions obtained from (13) and (6) into (12), we derive multiple solution cases contingent on the specified constraints. This procedure yields the following distinct cases:

**Case (1)**: If    $m_0=m_1=m_3=m_6=0$ then

$$s_{-1}=0 \,\ s_0=0 \,\ s_1=\pm \sqrt{\frac{2 \ m_4 \ (\gamma -\alpha )}{\beta }} \ ,\ \ \rho =\pm (2 \ a \ \alpha -\lambda ) \sqrt{\frac{b - a^2 \ (\alpha +r) - m_2 \ (\gamma -\alpha )}{r}}.$$

By systematically substituting the determined parameters into our solution framework, the solution to (1) in the form of a traveling wave is governed by:

**(1.1)** Under the conditions $m_2>0,\ m_4<0,$ and $\beta (\alpha -\gamma )>0$, the governing equation admits a bright soliton solution of the form:

14 $$\begin{aligned} V_{1.1}= \pm \sqrt{\frac{2\ m_2 (\alpha -\gamma )}{\beta }} \ \ \ \text {sech}\left[ (x-\lambda \ t)\ \sqrt{m_2} \right] \ e^{i \left( a x-b t+c_0\right) } . \end{aligned}$$

**(1.2)** Under the conditions $m_2<0, \ m_4>0,$ and $\beta \ (\alpha -\gamma )<0$, the governing equation admits a singular periodic solution:

15 $$\begin{aligned} V_{1.2}= \pm \sqrt{\frac{2 m_2 (\alpha -\gamma )}{\beta }} \ \sec \left[ (x-\lambda \ t) \ \sqrt{-m_2} \right] \ e^{i \left( a x-b t+c_0\right) } . \end{aligned}$$

**(1.3)** Under the conditions $m_2<0, m_4>0, and \ \beta (\alpha -\gamma )<0$, the governing equation admits a singular periodic solution:

16 $$\begin{aligned} V_{1.3}= \pm \sqrt{\frac{2 m_2 (\alpha -\gamma )}{\beta }} \ \csc \left[ (x-\lambda \ t) \ \sqrt{-m_2} \right] \ e^{i \left( a x-b t+c_0\right) }. \end{aligned}$$

**Case (2)**: If    $m_0=\frac{m_2^2}{4 m_4} \, \ m_1=m_3=m_6=0,$ then

- **(2.1)** $$ s_0=0 \ , \ s_1=0 \ , \ s_{-1}= \pm \sqrt{\frac{\gamma -\alpha }{2 \beta m_4}} m_2 \ , \ \rho =\pm (2 a \alpha -\lambda ) \sqrt{\frac{ b - a^2 (\alpha +r) - m_2 (\gamma -\alpha )}{r}} $$.
- **(2.2)** $$ s_0= 0 \, \ s_1= \pm \sqrt{\frac{2 m_4 (\gamma -\alpha )}{\beta }} \, \ s_{-1}= \pm \sqrt{\frac{\gamma -\alpha }{2 \beta m_4}} m_2 \, \ \rho =\pm \sqrt{\frac{(\lambda -2 a \alpha )^2 \left( b - a^2 (\alpha +r) + 2 m_2 (\gamma -\alpha )\right) }{r}} $$.
- **(2.3)** $$ s_0=0 \, \ s_1=\pm \sqrt{\frac{2 m_4 (\gamma -\alpha )}{\beta }} \, \ s_{-1}= \mp \sqrt{\frac{\gamma -\alpha }{2 \beta m_4} } m_2 \, \ \rho =\pm \sqrt{\frac{(\lambda -2 a \alpha )^2[ b - a^2 (\alpha +r) - 4 m_2 (\gamma -\alpha ) ] }{r}} $$.

By systematically substituting the determined parameters of (2.1) into our solution framework, the solution to (1) in the form of a traveling wave is governed by:

**(2.1.1)** Under the conditions $m_2<0, m_4>0, and \ \beta (\alpha -\gamma )<0$, the governing equation admits a singular soliton solution of the form:

17 $$\begin{aligned} V_{\text {2.1.1}}= \pm \sqrt{\frac{m_2 (\alpha -\gamma )}{\beta }} \ \coth \left[ (x-\lambda \ t) \ \sqrt{-\frac{m_2}{2}}\right] \ e^{i \left( a x-b t+c_0\right) } . \end{aligned}$$

**(2.1.2)** Under the conditions $m_2>0, m_4>0, and \ \beta (\gamma -\alpha )>0$, the governing equation admits a singular periodic solution of the form:

18 $$\begin{aligned} V_{\text {2.1.2}}= \pm \sqrt{\frac{m_2 (\gamma -\alpha )}{\beta }} \cot \left[ (x-\lambda \ t)\ \sqrt{\frac{m_2}{2}}\right] \ e^{i \left( a x-b t+c_0\right) }. \end{aligned}$$

By systematically substituting the determined parameters of (2.2) into our solution framework, the solution to (1) in the form of a traveling wave is governed by:

**(2.2.1)** Under the conditions $m_2<0, m_4>0, and \ \beta (\alpha -\gamma )<0$, the governing equation admits a singular soliton solution of the form:

19 $$\begin{aligned} V_{\text {2.2.1}} = \pm 2 \sqrt{\frac{m_2 (\alpha -\gamma )}{\beta }} \ \text {csch}\left[ (x-\lambda \ t)\ \sqrt{-2 m_2} \right] \ e^{i \left( a x-b t+c_0\right) }. \end{aligned}$$

**(2.2.2)** Under the conditions $m_2>0, m_4>0, and \ \beta (\gamma -\alpha )>0$, the governing equation admits a singular periodic solution of the form:

20 $$\begin{aligned} V_{\text {2.2.2}} = \pm 2\sqrt{\frac{m_2 (\gamma -\alpha )}{\beta }} \ \csc \left[ (x-\lambda \ t)\ \sqrt{2\ m_2} \right] \ e^{i \left( a x-b t+c_0\right) }. \end{aligned}$$

By systematically substituting the determined parameters of (2.3) into our solution framework, the solution to (1) in the form of a traveling wave is governed by:

**(2.3.1)** Under the conditions $m_2<0, m_4>0, and \ \beta (\alpha - \gamma )>0$, the governing equation admits a dark soliton solution of the form:

21 $$\begin{aligned} V_{\text {2.3.1}}= \pm \sqrt{\frac{m_2 (\alpha -\gamma )}{\beta }} \ \tanh \left( \sqrt{-\frac{m_2}{2}} (x-\lambda t)\right) \ e^{i \left( a x-b t+c_0\right) }. \end{aligned}$$

**(2.3.2)** Under the conditions $m_2>0, m_4>0, and \ \beta (\gamma -\alpha )>0$, the governing equation admits a singular periodic solution of the form:

22 $$\begin{aligned} V_{\text {2.3.2}}= \pm \sqrt{\frac{m_2 (\gamma -\alpha )}{\beta }} \ \tan \left[ (x-\lambda \ t)\ \sqrt{\frac{m_2}{2}}\right] \ e^{i \left( a x-b t+c_0\right) } . \end{aligned}$$

**Case (3)**: If    $m_3=m_4=m_6=0 \, \ s_1=0$ then

$$\begin{aligned} s_{-1}=\pm m_1 \sqrt{\frac{\gamma -\alpha }{2 \beta m_2}} \, \ s_0=\pm \sqrt{\frac{m_2 (\gamma -\alpha )}{2 \beta }} \, \ m_0=\frac{m_1^2}{4 m_2} \, \ \rho =\pm (2 a \alpha -\lambda ) \sqrt{\frac{2 b -2 a^2 (\alpha +r) -m_2 (\alpha -\gamma )}{2 r}}. \end{aligned}$$

By systematically substituting the determined parameters into our solution framework, the solution to (1) in the form of a traveling wave is governed by:

**(3.1)** Under the conditions $m_2>0, and \ \beta (\gamma -\alpha )>0$, the governing equation admits an exponential solution of the form:

23 $$\begin{aligned} V_{3.1}= \pm \sqrt{\frac{m_2 (\gamma -\alpha )}{2 \beta }} \left( 1-\frac{2 m_1}{m_1-2 m_2 e^{\sqrt{m_2} (x-\lambda t)}}\right) e^{i \left( a x-b t+c_0\right) } . \end{aligned}$$

**Case (4)**: If   $m_0=m_1=m_6=0 \,\ m_2=\frac{m_3^2}{4 m_4}$ then

$$\begin{aligned} s_{-1}=0 \, \ s_1=\pm \sqrt{\frac{2 m_4 (\gamma -\alpha )}{\beta }}\, \ s_0= \pm \ m_3 \sqrt{\frac{\gamma -\alpha }{8 \beta m_4}}\,\ \rho = \pm (2 a \alpha -\lambda ) \sqrt{\frac{m_2 (\gamma -\alpha )-2 \left( a^2 (\alpha +r)-b\right) }{2 r}}. \end{aligned}$$

By systematically substituting the determined parameters into our solution framework, the solution to (1) in the form of a traveling wave is governed by:

**(4.1)** Under the conditions $m_2>0, and \ \beta (\gamma -\alpha )>0$, the governing equation admits a dark soliton solution of the form:

24 $$\begin{aligned} V_{4.1}= -\sqrt{\frac{m_2(\gamma -\alpha )}{2 \beta }} \ \tanh \left( \frac{\sqrt{m_2}}{2} (x-\lambda t)\right) \ e^{i \left( a x-b t+c_0\right) }. \end{aligned}$$

**Case (5)**: If $m_2=m_4=m_6=0$ then

$$\begin{aligned} s_1&=0 \, \ s_{-1}=\pm \sqrt{\frac{2 m_0 (\gamma -\alpha )}{\beta }} \, \ s_0= \pm \root 3 \of {\frac{m_3}{m_0}} \ \sqrt{\frac{m_0 (\gamma -\alpha ) }{2 \beta }} \, \ m_1=-2 \root 3 \of { m_0^{2} \ m_3}\\ \rho&=\pm \sqrt{\frac{(\lambda -2 a \alpha )^2 \left( 2 b -2 a^2 (\alpha +r)-3 \root 3 \of {m_3^{2} \ m_0} (\alpha -\gamma )\right) }{2 r}} . \end{aligned}$$

By systematically substituting the determined parameters into our solution framework, the solution to (1) in the form of a traveling wave is governed by:

**(5.1)** Under the conditions $m_3>0, m_0>0, and \ \beta (\gamma -\alpha )>0$, the governing equation admits a Weierstrass elliptic solution of the form:

25 $$\begin{aligned} V_{5.1}=\pm \sqrt{\frac{\gamma -\alpha }{2 \beta }} \ \left( -\frac{m_1}{2 \sqrt{m_0}}-\frac{2 \sqrt{m_0}}{ \wp \left( \frac{\sqrt{m_3} (x-\lambda t)}{2} ,-\frac{4 m_1}{m_3},-\frac{4 m_1}{m_3}\right) }\right) \ e^{i \left( a x-b t+c_0\right) }. \end{aligned}$$

**Case(6)**: If $m_1=m_3=m_6=0$ then

**(6.1)** $$ s_1=0 \, \ s_0=0 \, \ s_{-1}=\pm \sqrt{\frac{2 m_0 (\gamma -\alpha )}{\beta }} \,\ \rho =\pm (2 a \alpha -\lambda ) \sqrt{\frac{b- a^2 (\alpha +r)-m_2 (\gamma -\alpha )}{r}} $$.

**(6.2)** $$ s_{-1}=0 \, \ s_0=0 \, \ s_1=\pm \sqrt{\frac{2 m_4 (\gamma -\alpha )}{\beta }} \, \ \rho =\pm (2 a \alpha -\lambda ) \sqrt{\frac{-\left( a^2 (\alpha +r)\right) +b-m_2 (\gamma -\alpha )}{r}} $$.

**(6.3)** $$ s_0=0 \, \ s_1=\pm \sqrt{\frac{2 m_4 (\gamma -\alpha )}{\beta }} \, \ s_{-1}=\pm \sqrt{\frac{2 m_0 (\gamma -\alpha )}{\beta }} \, \ \rho =\pm \sqrt{\frac{(\lambda -2 a \alpha )^2 \left( b -a^2 (\alpha +r)+\left( m_2-6 \sqrt{m_0} \sqrt{m_4}\right) (\alpha -\gamma )\right) }{r}} $$.

**(6.4)** $$ s_0=0 \, \ s_1=\pm \sqrt{\frac{2 m_4 (\gamma -\alpha )}{\beta }} \, \ s_{-1}=\mp \sqrt{\frac{2 m_0 (\gamma -\alpha )}{\beta }} \, \ \rho =\pm \sqrt{\frac{(\lambda -2 a \alpha )^2 \left( b -a^2 (\alpha +r) + \left( 6 \sqrt{m_0} \sqrt{m_4}+m_2\right) (\alpha -\gamma )\right) }{r}} $$.

By systematically substituting the determined parameters of (6.1) into our solution framework, the solution to (1) in the form of a traveling wave is governed by:

**(6.1.1)** Under the conditions $m_0=1, m_2=-p^2-1, m_4=p^2,\ \beta (\gamma -\alpha )>0 \ and \ 0\le p\le 1$, the governing equation admits a Jacobi elliptic (JE) solution of the form:

26 $$\begin{aligned} V_{\text {6.1.1}}=\pm \sqrt{\frac{2 (\gamma -\alpha )}{\beta }} \ \text {ns}(x-\lambda t) \ e^{i \left( a x-b t+c_0\right) } . \end{aligned}$$

By substituting p = 1 or p = 0 into (26), the system yields the following solutions, respectively, a singular soliton solution or singular periodic wave solutions:

27 $$\begin{aligned} V_{\text {6.1.1.1}}=\pm \sqrt{\frac{2 (\gamma -\alpha )}{\beta }} \coth (x-\lambda t) e^{i \left( a x-b t+c_0\right) }, \end{aligned}$$

or

28 $$\begin{aligned} V_{\text {6.1.1.2}}=\pm \sqrt{\frac{2 (\gamma -\alpha )}{\beta }} \ \csc (x-\lambda t) \ e^{i \left( a x-b t+c_0\right) }. \end{aligned}$$

**(6.1.2)** Under the conditions $m_0=-p^2, m_2=2 p^2-1, m_4=1-p^2,\ \beta (\alpha -\gamma )>0~ and~ \ 0<p\le 1$, the governing equation admits a JE solution of the form:

29 $$\begin{aligned} V_{\text {6.1.2}}= \pm \sqrt{\frac{2 p^2 (\alpha -\gamma )}{\beta }} \ \text {cn}(x-\lambda t) \ e^{i \left( a x-b t+c_0\right) }. \end{aligned}$$

By substituting p = 1 into (29), the system yields the following bright soliton:

30 $$\begin{aligned} V_{\text {6.1.2.1}}=\pm \sqrt{\frac{2 (\alpha -\gamma )}{\beta }} \ \text {sech}(x-\lambda t) \ e^{i \left( a x-b t+c_0\right) }. \end{aligned}$$

**(6.1.3)** Under the conditions $m_0=-1, m_2=2-p^2, m_4=p^2-1, \beta (\alpha -\gamma )>0 \ and \ 0\le p\le 1$, the governing equation admits a JE solution of the form:

31 $$\begin{aligned} V_{\text {6.1.3}}= \pm \sqrt{\frac{2 (\alpha -\gamma )}{\beta }} \ \text {dn}(x-\lambda t) \ e^{i \left( a x-b t+c_0\right) }. \end{aligned}$$

By substituting p = 1 into (31), the system yields the following bright soliton solution:

32 $$\begin{aligned} V_{\text {6.1.3.1}}=\pm \sqrt{\frac{2 (\alpha -\gamma )}{\beta }} \ \text {sech}(x-\lambda t) \ e^{i \left( a x-b t+c_0\right) }. \end{aligned}$$

**(6.1.4)** Under the conditions $m_0=1, m_2=2-4 p^2, m_4=1,\beta (\gamma -\alpha )>0 \ and \ 0\le p\le 1$ the governing equation admits a JE solution of the form:

33 $$\begin{aligned} V_{\text {6.1.4}}=\pm \sqrt{\frac{2 (\gamma -\alpha )}{\beta }} \ \text {cn}(x-\lambda t) \ \text {nd}(x-\lambda t) \ \text {ns}(x-\lambda t) \ e^{i \left( a x-b t+c_0\right) } . \end{aligned}$$

By substituting p = 1 or p = 0 into (33), the system yields the following solutions, respectively a singular soliton solution or singular periodic solutions:

34 $$\begin{aligned} V_{\text {6.1.4.1}}=\pm \sqrt{\frac{2 (\gamma -\alpha )}{\beta }} \coth (x-\lambda t) e^{i \left( a x-b t+c_0\right) }, \end{aligned}$$

or

35 $$\begin{aligned} V_{\text {6.1.4.2}}=\pm \sqrt{\frac{2 (\gamma -\alpha )}{\beta }} \cot (x-\lambda t) e^{i \left( a x-b t+c_0\right) }. \end{aligned}$$

**(6.1.5)** Under the conditions $m_0=-1, m_2=2-p^2, m_4=p^2-1,\beta (\alpha -\gamma )>0 \ and \ 0\le p < 1$ , the governing equation admits a JE solution of the form:

36 $$\begin{aligned} V_{\text {6.1.5}}= \pm \sqrt{\frac{2 (p-1)^2 (\gamma -\alpha )}{\beta }} \ \text {nc}(x-\lambda t) \ \text {nd}(x-\lambda t) \left( p \ \text {sn}(x-\lambda t)^2+1\right) e^{i \left( a x-b t+c_0\right) }. \end{aligned}$$

By substituting p = 0 into (36), the system yields the following singular periodic solution:

37 $$\begin{aligned} V_{\text {6.1.5.1}}=\pm \sqrt{\frac{2 ( \alpha -\gamma )}{\beta }} \ \sec (x-\lambda t) \ e^{i \left( a x-b t+c_0\right) }. \end{aligned}$$

By systematically substituting the determined parameters of (6.2) into our solution framework, the solution to (1) in the form of a traveling wave is governed by:

**(6.2.1)** Under the conditions $m_0=1, m_2=-p^2-1, m_4=p^2, \beta (\gamma -\alpha )>0 \ and \ 0 < p\le 1$ , the governing equation admits a JE solution of the form:

38 $$\begin{aligned} V_{\text {6.2.1}} = \pm \sqrt{\frac{2 p^2 (\gamma -\alpha )}{\beta }} \ \text {sn}(x-\lambda t) \ e^{i \left( a x-b t+c_0\right) }. \end{aligned}$$

By substituting p = 1 into (38), the system yields the following dark soliton solution:

39 $$\begin{aligned} V_{\text {6.2.1.1}} = \pm \sqrt{\frac{2 (\gamma -\alpha )}{\beta }} \ \tanh (x-\lambda t) \ e^{i \left( a x-b t+c_0\right) }. \end{aligned}$$

**(6.2.2)** Under the conditions $m_0=p^2-1, m_2=2-p^2,m_4=-1, \beta (\alpha - \gamma )>0 \ and \ 0 \le p\le 1$, the governing equation admits a JE solution of the form:

40 $$\begin{aligned} V_{\text {6.2.2}} = \pm \sqrt{\frac{2 (\alpha -\gamma )}{\beta }} \ \text {dn}(x-\lambda t) \ e^{i \left( a x-b t+c_0\right) }. \end{aligned}$$

By substituting p = 1 into (40), the system yields bright soliton solution:

41 $$\begin{aligned} V_{\text {6.2.2.1}} = \pm \sqrt{\frac{2 (\alpha -\gamma )}{\beta }} \ \text {sech}(x-\lambda t) \ e^{i \left( a x-b t+c_0\right) }. \end{aligned}$$

**(6.2.3)** Under the conditions $m_0=-p^2, m_2=2 p^2-1, m_4=1-p^2, \beta (\alpha - \gamma )>0 \ and \ 0 \le p < 1$ , the governing equation admits a JE solution of the form:

42 $$\begin{aligned} V_{\text {6.2.3}} = \pm \sqrt{\frac{2 \left( p^2-1\right) (\alpha -\gamma )}{\beta }} \ \text {nc}(x-\lambda t) \ e^{i \left( a x-b t+c_0\right) }. \end{aligned}$$

By substituting p = 0 into (42), the system yields the following singular periodic solution:

43 $$\begin{aligned} V_{\text {6.2.3.1}} = \pm \sqrt{\frac{2 (\gamma -\alpha )}{\beta }} \ \sec (x-\lambda t) \ e^{i \left( a x-b t+c_0\right) }. \end{aligned}$$

**(6.2.4)** Under the conditions $m_0=1, m_2=2-4 p^2, m_4=1, \beta (\gamma - \alpha )>0 \ and \ 0 \le p \le 1$ , the governing equation admits a JE solution of the form:

44 $$\begin{aligned} V_{\text {6.2.4}} = \pm \sqrt{\frac{2 (\gamma -\alpha )}{\beta }} \ \text {dn}(x-\lambda t) \ \text {nc}(x-\lambda t) \ \text {sn}(x-\lambda t) \ e^{i \left( a x-b t+c_0\right) }. \end{aligned}$$

By substituting p = 1 or p = 0 into (44), the system yields the following solutions, respectively a dark soliton solution or singular periodic solution :

45 $$\begin{aligned} V_{\text {6.2.4.1}} = \pm \sqrt{\frac{2 (\gamma -\alpha )}{\beta }} \ \tanh (x-\lambda t) \ e^{i \left( a x-b t+c_0\right) }, \end{aligned}$$

or

46 $$\begin{aligned} V_{\text {6.2.4.2}} = \pm \sqrt{\frac{2 (\gamma -\alpha )}{\beta }} \ \tan (x-\lambda t) \ e^{i \left( a x-b t+c_0\right) }. \end{aligned}$$

**(6.2.5)** Under the conditions $m_0=p^4-2 p^3+p^2, m_2=-\frac{4}{p},$ $m_4=-p^2+6 p-1, \beta$ $\left( p^2-6 p+1\right) (\alpha -\gamma )>0 \ and \ 0<p\le 1$ , the governing equation admits a JE solution of the form:

47 $$\begin{aligned} V_{\text {6.2.5}} = \pm \sqrt{\frac{2 \left( p^2-6 p+1\right) (\alpha -\gamma )}{\beta }} \ \dfrac{p \ \text {cn}(x-\lambda t) \ \text {dn}(x-\lambda t)}{p \ \text {sn}(x-\lambda t)^2+1} \ e^{i \left( a x-b t+c_0\right) }. \ \end{aligned}$$

By substituting p = 1 into (47), the system yields the following bright soliton solution:

48 $$\begin{aligned} V_{\text {6.2.5.1}} = \pm \sqrt{\frac{8 (\gamma -\alpha )}{\beta }} \ \text {sech}(2 (x-\lambda t)) \ e^{i \left( a x-b t+c_0\right) }. \end{aligned}$$

By systematically substituting the determined parameters of (6.3) into our solution framework, the solution to (1) in the form of a traveling wave is governed by:

**(6.3.1)** Under the conditions $m_0=1, m_2=-p^2-1, m_4=p^2,\beta (\gamma - \alpha )>0 \ and \ 0 \le p \le 1$ , the governing equation admits a JE solution of the form:

49 $$\begin{aligned} V_{\text {6.3.1}} = \pm \sqrt{\frac{2 (\gamma -\alpha )}{\beta }} \ \text {ns}(x-\lambda t) \left( p \ \text {sn}(x-\lambda t)^2-1\right) \ e^{i \left( a x-b t+c_0\right) }. \end{aligned}$$

By substituting p = 1 or p = 0 into (49), the system yields the following solutions, respectively a singular soliton solution or singular periodic solution:

50 $$\begin{aligned} V_{\text {6.3.1.1}} = \mp \sqrt{\frac{8 (\gamma -\alpha )}{\beta }} \ \text {csch}(2 (x-\lambda t)) \ e^{i \left( a x-b t+c_0\right) }, \end{aligned}$$

or

51 $$\begin{aligned} V_{\text {6.3.1.2}} = \mp \sqrt{\frac{2 (\gamma -\alpha )}{\beta }} \ \csc (x-\lambda t) \ e^{i \left( a x-b t+c_0\right) } . \end{aligned}$$

**(6.3.2)** Under the conditions $m_0=p^2-1, m_2=2-p^2, m_4=-1, \beta (\alpha -\gamma )>0 \ and \ 0 \le p \le 1$, the governing equation admits a JE solution of the form:

52 $$\begin{aligned} V_{\text {6.3.2}} = \pm \sqrt{\frac{2 (\alpha -\gamma )}{\beta }} \left( \frac{\sqrt{1-p^2}}{\text {dn}(x-\lambda t)}+\text {dn}(x-\lambda t)\right) e^{i \left( a x-b t+c_0\right) }. \end{aligned}$$

By substituting p = 1 into (52), the system yields the following bright soliton solution:

53 $$\begin{aligned} V_{\text {6.3.2.1}} = \pm \sqrt{\frac{2 (\alpha -\gamma )}{\beta }} \ \text {sech}(x-\lambda t) \ e^{i \left( a x-b t+c_0\right) }. \end{aligned}$$

**(6.3.3)** Under the conditions $m_0=-p^2, m_2=2 p^2-1, m_4=1-p^2, \beta (\alpha -\gamma )>0 \ and \ 0 \le p \le 1$, the governing equation admits a JE solution of the form:

54 $$\begin{aligned} V_{\text {6.3.3}} = \pm \sqrt{\frac{2 (\alpha -\gamma )}{\beta }} \ \text {cn}(x-\lambda t) \left( \sqrt{p^2-1} \ \text {nc}(x-\lambda t)^2+p\right) \ e^{i \left( a x-b t+c_0\right) } . \end{aligned}$$

By substituting p = 1 or p = 0 into (54), the system yields the following solutions, respectively a bright soliton solution and singular periodic solution:

55 $$\begin{aligned} V_{\text {6.3.3.1}} = \pm \sqrt{\frac{2 (\alpha -\gamma )}{\beta }} \ \text {sech}(x-\lambda t) \ e^{i \left( a x-b t+c_0\right) }, \end{aligned}$$

or

56 $$\begin{aligned} V_{\text {6.3.3.2}} = \pm \sqrt{\frac{2 (\gamma -\alpha )}{\beta }} \ \sec (x-\lambda t) \ e^{i \left( a x-b t+c_0\right) }. \end{aligned}$$

**(6.3.4)** Under the conditions $m_0=1, m_2=2-4 p^2, m_4=1, \beta (\gamma - \alpha )>0 \ and \ 0 \le p \le 1$, the governing equation admits a JE solution of the form:

57 $$\begin{aligned} V_{\text {6.3.4}} = \pm \sqrt{\frac{2 (\gamma -\alpha )}{\beta }} \ \frac{\ \left( \text {dn}(x-\lambda t)^2 \ \text {nc}(x-\lambda t)^2 \ \text {sn}(x-\lambda t)^2+1\right) \text {cn}(x-\lambda t) }{\text {dn}(x-\lambda t) \ \text {sn}(x-\lambda t)} \ e^{i \left( a x-b t+c_0\right) }. \end{aligned}$$

By substituting p = 1 or p = 0 into (57), the system yields the following solutions, respectively a singular soliton solution or singular periodic solution:

58 $$\begin{aligned} V_{\text {6.3.4.1}} = \pm \sqrt{\frac{8 (\gamma -\alpha )}{\beta }} \ \coth (2 (x-\lambda t)) \ e^{i \left( a x-b t+c_0\right) }, \end{aligned}$$

or

59 $$\begin{aligned} V_{\text {6.3.4.2}} = \pm \sqrt{\frac{8 (\gamma -\alpha )}{\beta }} \ \csc (2 (x-\lambda t)) \ e^{i \left( a x-b t+c_0\right) }. \end{aligned}$$

**(6.3.5)** Under the conditions $m_0=p^4-2 p^3+p^2, m_2=-\frac{4}{p},$ $m_4=-p^2+6 p-1, \beta (\gamma - \alpha )>0,$ $and \ 0 \le p \le 1$, the governing equation admits a JE solution of the form:

60 $$\begin{aligned} V_{\text {6.3.5}} = \pm \sqrt{\frac{2 (\alpha - \gamma )}{\beta }} \ \left( \frac{p \ \sqrt{p^2-6 p+1} \ \text {cn}(x-\lambda t)^2 \ \text {dn}(x-\lambda t)^2+\sqrt{-(p-1)^2} \left( \text {dn}(x-\lambda t)^2-2\right) ^2}{\text {cn}(x-\lambda t) \ \text {dn}(x-\lambda t) \left( p \ \text {sn}(x-\lambda t)^2+1\right) } \right) \end{aligned}$$

   $$ \times \ e^{i \left( a x-b t+c_0\right) }. $$

By substituting p = 1 or p = 0 into (60), the system yields the following solutions, respectively a singular soliton solution or singular periodic solution:

61 $$\begin{aligned} V_{\text {6.3.5.1}} = \pm \sqrt{\frac{8 (\gamma -\alpha )}{\beta }} \ \text {sech}(2 (x-\lambda t)) \ e^{i \left( a x-b t+c_0\right) }, \end{aligned}$$

or

62 $$\begin{aligned} V_{\text {6.3.5.2}} = \pm \sqrt{\frac{2 (\gamma -\alpha )}{\beta }} \ \sec (x-\lambda t) \ e^{i \left( a x-b t+c_0\right) }. \end{aligned}$$

## Modulational instability analysis in the MCGLE

The study of modulational instability (MI) in complex nonlinear systems provides critical insights into wave propagation dynamics, particularly when dispersion and nonlinearity interact to destabilize coherent structures. For the modified complex Ginzburg-Landau equation (MCGLE) considered here, MI analysis reveals how small perturbations evolve in the presence of higher-order nonlinearities and complex coefficients. We examine perturbed solutions of the MCGLE (1) by introducing a complex modulation around the steady state:

63 $$\begin{aligned} V= \left( \mathcal {H}(x,t)+\sqrt{\mathcal {A}}\right) \ e^{i (\mathcal {A} t)}, \end{aligned}$$

where $\mathcal {A}$ represents the normalized background power, and $\mathcal {H}(x,t)$ is a complex perturbation. Substituting into (1) and linearizing yields the complex evolution equation for H:

64 $$\begin{aligned} \left( \mathcal {H}^*+\mathcal {H}\right) \left( 4 \mathcal {A}^2 \beta -2 \mathcal {A}^2-2 i \mathcal {A} \rho \right) +i \mathcal {A} \mathcal {H}_t+\mathcal {H}_{\text {xx}} (\alpha \mathcal {A}-\mathcal {A} \gamma )=0, \end{aligned}$$

where * indicates the conjugate of complex function. To resolve the complex frequency response, we assume a plane-wave perturbation:

65 $$\begin{aligned} \mathcal {H}=f_1 e^{i (L x-t \omega )}+f_2 e^{-i (L x-t \omega )}, \end{aligned}$$

where L $\in$ R is the wavenumber, and $\omega$ $\in$ C is the complex frequency:

66 $$\begin{aligned} \omega =L \left( \sqrt{\frac{\left( \sqrt{Q^2+Y^2}+Q\right) }{2} } + i \ \text {sgn}(Y) \ \sqrt{\frac{\left( \sqrt{Q^2+Y^2}-Q\right) }{2} } \ \right) , \end{aligned}$$

where

$$\begin{aligned} Q = (\alpha -\gamma ) \left( \mathcal {A} (4-8 \beta )+L^2 (\alpha -\gamma )\right) , \ and \ Y = 4 \ \rho \ (\alpha -\gamma ). \end{aligned}$$

The stability characteristics of the system are determined by analyzing the complex perturbation frequency $\omega = \omega _r + i\omega _i$.

- **Exponential Stability:** When $\omega _i > 0$, perturbations decay exponentially over time, preserving the system’s stable solutions.
- **Modulational Instability (MI):** When $\omega _i < 0$, small disturbances grow exponentially, leading to wave pattern disintegration.
- **Neutral Stability:** When $\omega _i = 0$ with $\omega _r \ne 0$, the system exhibits sustained oscillatory behavior without amplitude growth or decay.
- **Purely Exponential Dynamics:** When $\omega _r = 0$, the system evolves non-oscillatorily through either exponential growth ($\omega _i < 0$) or decay ($\omega _i > 0$).

**Fig. 1:**
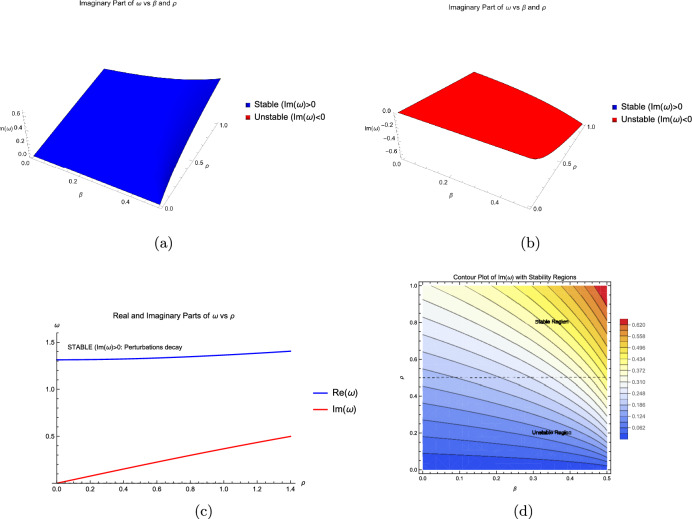
Graphical representation of stability analysis.

The imaginary component $\omega _i$ governs amplitude dynamics, while the real component $\omega _r$ regulates oscillatory behavior. In our specific analysis (Fig. 1), the growth rate of modulational instability is directly quantified by $-\omega _i$ for $\omega _i < 0$. The parameters used in Fig. 1 correspond to distinct physical regimes: Curve A ($\alpha =1.0, \gamma =0.5, \rho =0.1$) demonstrates the classical Benjamin-Feir instability with maximum growth at finite wavenumber; Curve B ($\alpha =1.0, \gamma =2.0, \rho =0.3$) shows enhanced instability bandwidth; Curve C ($\alpha =-0.5, \gamma =0.5, \rho =0.05$) exhibits shifted instability bands due to anomalous dispersion. This quantitative framework connects directly with established CGL literature while highlighting features specific to the modified equation.

## Result and discussion

**Fig. 2:**
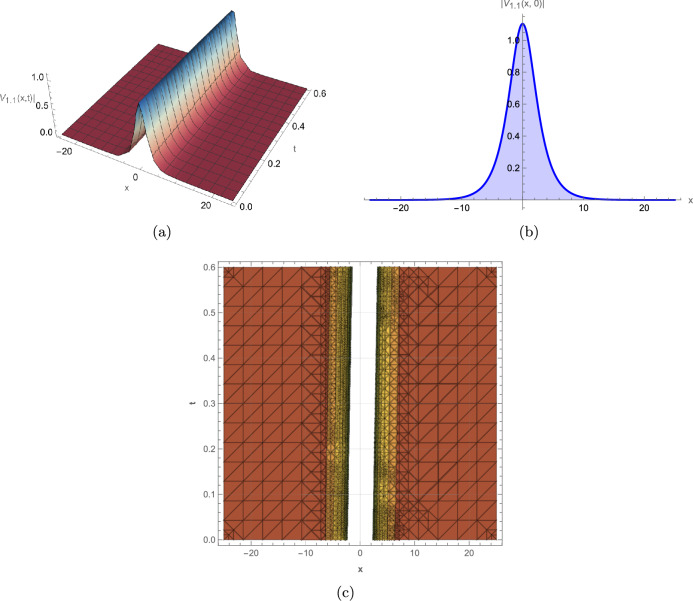
Graphical representation of the bright soliton solution of (13).

**Fig. 3:**
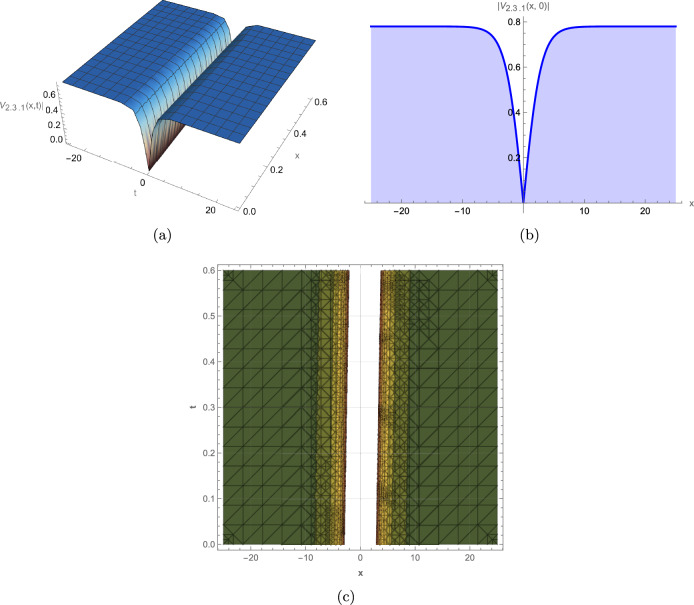
Graphical representation of the dark soliton solution of (20).

**Fig. 4:**
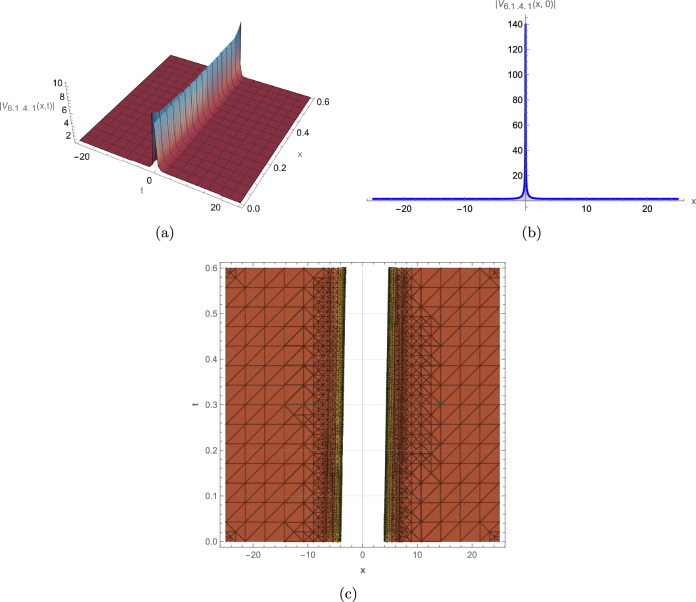
Graphical representation of the singular soliton solution of (33).

**Fig. 5:**
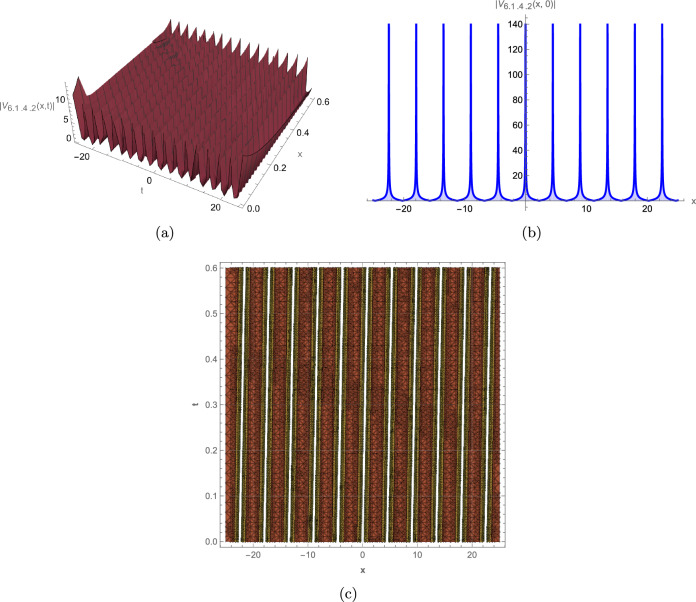
Graphical representation of the singular periodic solution of (34).

**Fig. 6:**
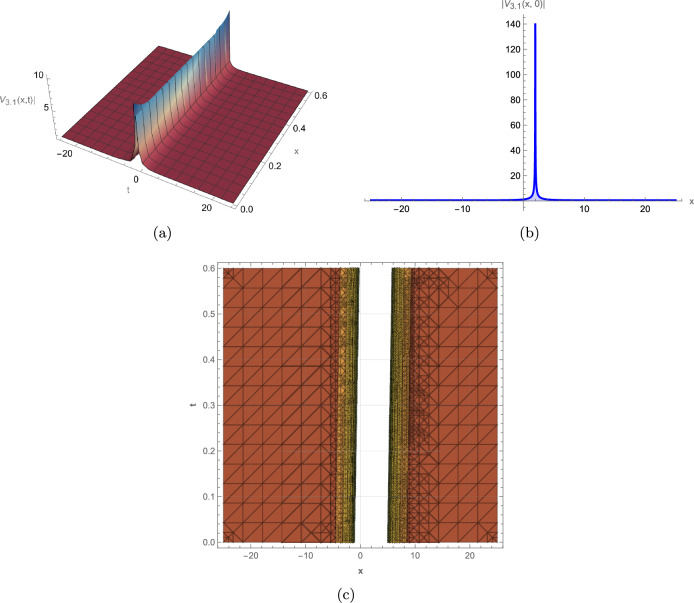
Graphical representation of the exponential solution of (22).

This study successfully derives a new family of exact wave solutions for the modified complex Ginzburg-Landau equation (MCGLE) by employing the modified extended direct algebraic method. The obtained solutions demonstrate a remarkable diversity of wave structures, encompassing bright and dark solitons, singular solitons, periodic waves, exponential solutions, and sophisticated Weierstrass elliptic function solutions. The obtained wave profiles are consistent with recent dynamical analyses observed in other nonlinear models, such as the dispersive solitons investigated by Bilal et al.^31^ and the hybrid structures reported by Ismael et al.^32^. Each family of solutions obtained in this study corresponds to distinct physical phenomena observed in nonlinear dispersive media: **Bright Solitons:** These localized, particle-like waves maintain constant shape and velocity due to exact balance between nonlinear self-focusing and anomalous dispersion. They represent stable wave packets that propagate without distortion, making them ideal carriers for optical communication bits and models for stable coherent structures in Bose-Einstein condensates. **Dark Solitons:** Characterized by intensity dips on a continuous background with associated phase jumps, these solutions arise from balance between defocusing nonlinearity and normal dispersion. They exhibit remarkable stability against perturbations and noise, serving as robust information carriers in optical fibers and representing vortex filaments in fluid dynamics. **Singular Solitons:** These solutions feature finite-time or finite-space singularities where wave amplitude becomes locally unbounded. They model critical phenomena such as wave collapse in nonlinear optics, rogue wave formation in oceans, and focusing singularities in plasma physics, providing insights into system instability thresholds. **Periodic Solutions:** Representing continuous wave trains with regular repetition, these solutions model mode-locked laser outputs, Brillouin scattering patterns, and crystal lattice vibrations. Their spectral purity makes them essential for frequency comb generation and high-precision metrology. **Weierstrass Elliptic Function Solutions:** These doubly periodic solutions generalize standard periodic waves, capturing more complex interference patterns in nonlinear media. They describe nonlinear wave interactions in periodic potentials, photonic crystals, and superlattice structures where multiple spatial periods coexist. **Exponential Solutions:** Characterized by monotonic growth or decay, these non-oscillatory profiles model evanescent waves, boundary layer effects, and dissipative processes where energy exchange dominates over wave propagation. They are crucial for understanding surface phenomena, tunneling effects, and absorption processes. To elucidate the physical characteristics and dynamical behavior of these solutions, we provide comprehensive graphical visualizations including 3D surface plots, 2D profiles, and contour maps of intensity distribution. These visualizations are instrumental in deciphering the complex spatiotemporal evolution and stability properties of optical solitons within nonlinear media. The modified complex Ginzburg-Landau equation (MCGLE) is governed by the interaction of key physical parameters: $\alpha$ (group velocity dispersion, GVD) controls pulse broadening; $\beta$ (self-phase modulation, SPM) provides nonlinear balancing; $\gamma$ represents higher-order dispersion effects; and $\rho$ accounts for nonlinear saturation. The delicate balance among these parameters determines the stability and morphology of the resulting wave structures. Figure 2: Bright Soliton exhibits a characteristic hyperbolic secant profile with maximum intensity at its center. The symmetric, localized structure maintains constant amplitude and width during propagation, confirming perfect balance between anomalous dispersion ($\alpha > 0$) and self-focusing nonlinearity ($\beta > 0$). The contour plot reveals straight, parallel intensity contours, indicating uniform velocity and absence of radiative decay–essential features for distortion-free signal transmission in optical fibers. The robustness of this structure under parameter variation suggests its potential as an information carrier in long-haul communication systems. Figure 3: Dark Soliton displays a characteristic intensity dip on a non-zero background with an associated $\pi$-phase shift across its center. The stable propagation of this “hole” solution arises from the balance between normal dispersion ($\alpha < 0$) and defocusing nonlinearity. The depth and width of the dip are governed by the ratio $|\alpha /\beta |$, while the steepness of the intensity gradient correlates with the saturation parameter $\rho$. Such structures are particularly resistant to noise-induced distortions, making them suitable for precision metrology and dark-pulse-based communication schemes. Figure 4: Singular Soliton features sharply peaked, non-diverging structures where intensity reaches local maxima without blowing up. The finite singularity results from controlled nonlinear focusing overcoming dispersive spreading, with the parameter $\gamma$ (higher-order dispersion) regulating the peak sharpness. These solutions represent critical states near instability thresholds and model phenomena like rogue waves or extreme pulse compression events in nonlinear waveguides. Figure 5: Singular Periodic Solution combines localized singular peaks with underlying periodicity. The periodic modulation wavelength is determined by the phase parameter $\lambda$, while the singular peak spacing correlates with the wave number *k*. This hybrid structure illustrates the competition between periodic dispersion waves and localized nonlinear focusing–a behavior observed in mode-locked lasers generating pulse trains and in plasma wave interactions. Figure 6: Exponential Solution shows monotonic decay/growth profiles characteristic of dissipative dominated regimes. The decay rate is exponentially proportional to the ratio $|\gamma /\alpha |$, reflecting the dominance of loss/gain mechanisms over dispersion. Such profiles model boundary layer phenomena, evanescent waves in photonic bandgap materials, and the penetration depth of surface plasmon polaritons. The systematic variation of parameters reveals critical transitions between solution types: increasing $\beta$ enhances nonlinear localization, leading to brighter solitons; positive $\gamma$ values promote higher-order wave shaping; while $\rho$ moderates extreme nonlinearities, preventing collapse. These graphical analyses provide intuitive guidelines for tailoring wave structures in practical applications by strategically tuning physical parameters in nonlinear optical devices, plasma confinement systems, and hydrodynamic wave models.

### Detailed solution analysis

For instance, Fig. 2 depicts a robust bright soliton solution, generated with the parameters $\alpha =0.9, \gamma =0.45$, $\beta =0.37, m_2=0.5$, $a=0.6, b=-0.5$, $c_0=0.8, \lambda =0.7$. This localized wave packet maintains its shape and amplitude during propagation, a hallmark of soliton behavior, highlighting its potential for stable information transfer in optical systems. Figure 3 illustrates a dark soliton solution, generated with parameters $\alpha =0.45, \gamma =0.9$, $\beta =0.37, m_2=-0.5$, $a=0.6, b=-0.5$, $c_0=0.8, \lambda =0.7$. Unlike the bright soliton, the dark soliton maintains a notch-like structure with a distinct phase shift across the dip. This wave remains stable during propagation because of a balance between dispersion and nonlinearity, making it crucial for optical communication systems where phase control is critical. Figure 4 represents a singular soliton solution generated with parameters $\alpha =0.45, \gamma =0.9$, $\beta =0.37,a=0.6$, $b=-0.5,c_0=0.8,\lambda =0.7$. This solution emerges when nonlinear focusing overcomes dispersive spreading, creating localized intensity peaks that remain finite due to higher-order stabilization effects. Figure 5 depicts a singular periodic solution, generated with parameters $\alpha =0.45,\gamma =0.9$, $\beta =0.37$, $a=0.6,b=-0.5$, $c_0=0.8,\lambda =0.7$. This type of solution integrates periodic oscillatory behavior with singular features, reflecting complex interactions between nonlinearity and dispersion in physical systems such as fluid dynamics, plasma waves, or optical fibers. Figure 6 represents an exponential solution generated with parameters $\alpha =0.45,\gamma =0.9$, $\beta =0.37,m_2=0.5$, $m_1=0.38,a=0.6$, $b=-0.5,c_0=0.8,\lambda =0.7$. This solution is distinguished by a non-oscillatory waveform that decays exponentially, modeling energy dissipation processes and boundary layer phenomena in applied physics contexts.

The obtained analytical solutions provide significant application in several physical domains. Bright solitons behave as robust information carriers in long-distance fiber optics by resisting dispersion

## Conclusion

Through the systematic application of the modified extended direct algebraic method (MEDAM), this investigation has successfully uncovered a rich spectrum of exact analytical solutions for the modified complex Ginzburg-Landau equation. The primary novelty of this work lies in the derivation of advanced solution families–such as Jacobi and Weierstrass elliptic function solutions–that had not been previously reported for this specific model, extending beyond the basic trigonometric and hyperbolic solutions found in earlier studies^28^. Additionally, we obtained dark, bright, and singular solitons, along with periodic and exponential wave solutions. All solutions were rigorously characterized through comprehensive 3D surface visualizations, 2D profile analyses, and density plot mappings to elucidate their nonlinear dynamics. Crucially, a detailed modulational instability analysis (Section “Modulational instability analysis in the MCGLE”) was performed to assess the stability properties of these wave solutions under various parameter conditions, providing essential insights into their physical robustness and viability. The usefulness and practical significance of these findings are threefold. First, they advance the fundamental understanding of nonlinear wave propagation in dissipative systems, providing a broader set of exact benchmarks for validating numerical simulations. Second, the stability analysis offers critical predictive capability for determining parameter regimes where stable wave propagation can be maintained or where instability-induced pattern formation may occur. Third, the demonstrated efficacy of MEDAM coupled with stability assessment offers a powerful analytical framework that can be adapted to other complex nonlinear systems in fields such as nonlinear optics, plasma physics, and condensed matter theory, where precise modeling and stability control of wave phenomena are critical. These findings pave the way for several promising future research directions, including explorations of multi-component coupled systems with cross-phase modulation, stochastic formulations incorporating multiplicative noise effects, fractional-order generalizations capturing anomalous dispersion phenomena, and data-driven approaches combining machine learning with symbolic computation for solution discovery in higher-dimensional settings. While MEDAM proved highly effective for this study, certain methodological considerations exist. The approach relies on a balance between dispersion and nonlinearity and is most naturally suited for deriving traveling wave solutions. For analyzing transient dynamics or highly non-integrable systems in higher dimensions, complementing MEDAM with numerical techniques is recommended.

## Data Availability

The datasets used and/or analysed during the current study are available from the corresponding author on reasonable request.
